# Effect of Microencapsulated Temperature Rise Inhibitor on the Temperature Rise of Medium-Sized Concrete

**DOI:** 10.3390/ma18061230

**Published:** 2025-03-10

**Authors:** Yingda Zhang, Junru Zhang, Jun Chen, Zhijian Yan, Xinyue Liu, Haojie Zhang

**Affiliations:** 1 Key Laboratory of Transportation Tunnel Engineering, Ministry of Education, School of Civil Engineering, Southwest Jiaotong University, Chengdu 610031, China; yingda.zhang@xhu.edu.cn (Y.Z.);; 2 School of Architecture and Civil Engineering, Xihua University, Chengdu 610039, China; 3 Jinhua Xinsheng Zeolite Development Co., Ltd., Jinhua 321000, China

**Keywords:** microencapsulated TRI, hydration temperature, medium-sized concrete, thermal cracking, XRD, SEM, optimal dosage

## Abstract

This study investigates the effect of microencapsulated temperature rise inhibitors (TRIs) on the hydration temperature evolution and crack resistance of medium-sized concrete structures. Unlike mass concrete, medium-sized concrete elements such as beams, slabs, and columns pose unique challenges in temperature control due to their moderate volume, limited heat dissipation, and susceptibility to thermal stress-induced cracking. To address this issue, concrete mixtures with TRI dosages of 0%, 0.05%, 0.1%, and 0.15% were evaluated using a sealed foam box method, allowing for precise monitoring of hydration temperature development under insulated conditions. The results indicate that TRIs effectively suppress peak hydration temperature and delays its occurrence, with higher TRI dosages leading to more pronounced effects. X-ray diffraction (XRD) and scanning electron microscopy (SEM) analyses confirm that the hydration suppression is attributed to a controlled-release mechanism, where TRIs gradually dissolve, forming a hydration barrier on cement particles. This slows down calcium hydroxide (CH) crystallization, alters C-S-H gel evolution, and reduces early age heat accumulation, mitigating thermal cracking risks. Furthermore, mechanical property tests reveal that, while early age compressive and tensile strength decrease with TRI addition, long-term strength recovery is achieved at optimum TRI dosages. This study identifies 0.1% TRI as the most effective dosage, striking a balance between hydration heat reduction and long-term mechanical performance. These findings provide a scientific basis for optimizing TRI dosages in medium-sized concrete applications, offering a practical solution for thermal cracking prevention.

## 1. Introduction

Modern concrete engineering plays a crucial role in infrastructure development, with widespread applications in bridges, tunnels, underground structures, and high-rise buildings [1]. However, one of the major challenges in concrete construction is managing the heat generated during the hydration process. As cement reacts with water, a significant amount of heat is released, leading to a rapid rise in internal temperature [2]. If the temperature gradient between the interior and surface of concrete becomes excessive, thermal stresses are induced, which can result in thermal cracking. These cracks not only compromise the mechanical integrity of the structure but also accelerate deterioration, reducing durability and service life [3]. Effective temperature control measures are therefore essential to ensure the long-term performance of concrete structures [4].

Extensive research has been conducted on mass concrete, where controlling hydration heat is particularly critical due to its large volume and slow heat dissipation [5]. Various temperature control strategies, including cooling pipe systems [6], optimized mix design [7], and the use of supplementary cementitious materials [8], have been developed to mitigate thermal cracking in mass concrete applications. However, medium-sized concrete structures, such as slabs, beams, and columns, also face significant risks of temperature-induced cracking. These components, despite their relatively smaller dimensions, often have inadequate heat dissipation, especially in environments with poor ventilation or external constraints [9]. Despite their prevalence in construction, the thermal behavior of medium-sized concrete and its associated cracking risks have received considerably less attention in research, leaving a critical gap in the current understanding of temperature control in such structures.

Predicting temperature-induced cracking and microstructural degradation in concrete structures has been a significant focus in recent research. Various numerical approaches have been developed to model the fracture mechanics and thermal damage in concrete. For instance, cohesive crack models have been widely used to simulate the degradation of dynamic properties in concrete under mixed-mode fracture conditions [10]. Additionally, microencapsulation-based self-healing mechanisms have shown promise in mitigating damage and inhibiting chloride–water coupled transport in concrete. These numerical insights provide valuable frameworks for understanding how microencapsulated temperature rise inhibitors (TRIs) influence thermal cracking behavior [11].

To address the issue of thermal cracking in concrete structures, various temperature control strategies have been developed. One widely adopted approach is material optimization, which involves using low-heat cement or incorporating supplementary cementitious materials (SCMs), such as fly ash and ground granulated blast-furnace slag (GGBS) [12,13]. These materials reduce the heat of hydration by partially replacing cement, thereby lowering the peak temperature during the hydration process. Another commonly used method is external cooling, where cooling pipe systems are embedded within mass concrete structures to dissipate excess heat through circulating water [14]. This technique effectively controls temperature rise but is often complex and costly to implement. Additionally, chemical admixtures, such as water-reducing agents and retarders, have been utilized to modify hydration kinetics, delaying peak temperature occurrence and mitigating thermal stress development [15]. While these strategies have proven effective in large-scale applications, their applicability to medium-sized concrete structures remains uncertain.

Despite the extensive research on temperature control in mass concrete, there is a significant research gap regarding medium-sized concrete. Cooling pipe systems, while effective in mass concrete, are impractical for medium-sized elements due to their moderate volume and construction constraints. This highlights the need for alternative solutions tailored to medium-sized concrete. In recent years, temperature rise inhibitors (TRIs) have emerged as a promising admixture-based approach to controlling hydration heat [16]. These inhibitors work by regulating cement hydration reactions, reducing the maximum temperature rise, and delaying heat generation without compromising long-term mechanical performance.

There are various types of TRIs available, including urea, sugars, phase change materials (PCMs), and microcapsules. Each material has its own advantages and disadvantages. For instance, urea offers excellent cooling effects and minimal shrinkage, but it also results in high early age porosity, low strength, and durability risks associated with high dosages [17]. PCMs provide a gentle temperature increase and decrease curve, yet some PCMs may increase porosity and reduce mechanical properties [18]. Sugars and their derivatives have a low early hydration rate with no long-term effects, but they can significantly extend the setting time [19]. Microcapsules offer good hydration control; however, excessive dosages can affect the setting time [20]. In view of the above studies, it can be found that microcapsules can regulate the rise in concrete hydration temperature without adverse effects on the concrete itself, representing an effective and novel temperature- suppressing material. However, research on the application of TRIs in medium-sized concrete structures remains limited, and their effectiveness under varying dosage levels is not well understood. Therefore, experimental validation is necessary to systematically evaluate the influence of different TRI dosages on hydration temperature rise and concrete performance, providing a scientific basis for optimizing temperature control strategies in medium-sized concrete applications.

Unlike previous studies that primarily focused on large-scale concrete structures, this study investigates the application of TRIs in medium-sized concrete elements, such as beams and slabs, where heat dissipation and thermal stress characteristics differ from mass concrete. The controlled-release mechanism of microencapsulated TRIs in medium-sized elements has not been extensively explored. This research provides a comprehensive experimental evaluation of optimal TRI dosages for mitigating thermal cracking while maintaining mechanical performance in medium-sized concrete applications.

Traditional methods for evaluating the heat release characteristics of cement hydration, such as direct hydration tests, microcalorimetry, and adiabatic temperature rise tests, often involve complex operations, extended testing durations, and limited batch testing capabilities [21,22,23]. In contrast, the foam box method offers a simplified, stable, and scalable approach for assessing hydration temperature rise in concrete. The use of insulated foam boxes effectively minimizes external heat loss, ensuring that the recorded temperature primarily reflects internal hydration reactions rather than environmental influences. Additionally, this method allows for batch testing, improving experimental efficiency and repeatability. By providing a controlled thermal environment, the foam box system enables a more realistic simulation of in situ concrete conditions, particularly for medium-sized concrete elements, where external cooling measures are impractical. Due to its simplicity and reliability, this approach has been formally incorporated into the Chinese Standard T/CECS 10270-2023 for temperature control evaluation of concrete materials, further validating its effectiveness in practical applications [24].

This study aims to systematically investigate the effect of microencapsulated TRI dosage (0%, 0.05%, 0.1%, and 0.15%) on the hydration temperature rise in medium-sized concrete structures. Given the limited research on thermal control in such structures, this study focuses on experimentally analyzing how different TRI dosages influence peak hydration temperature and heating rate using the simplified foam box test method. By evaluating these key parameters, the research seeks to determine the optimal microencapsulated TRI dosage that effectively mitigates temperature-induced cracking while maintaining concrete performance. Moreover, this study integrates X-ray diffraction (XRD) and scanning electron microscopy (SEM) to provide in-depth insights into microstructural changes induced by TRI incorporation.

## 2. Experimental Program

### 2.1. Materials and Mix Design

**Figure 1:**
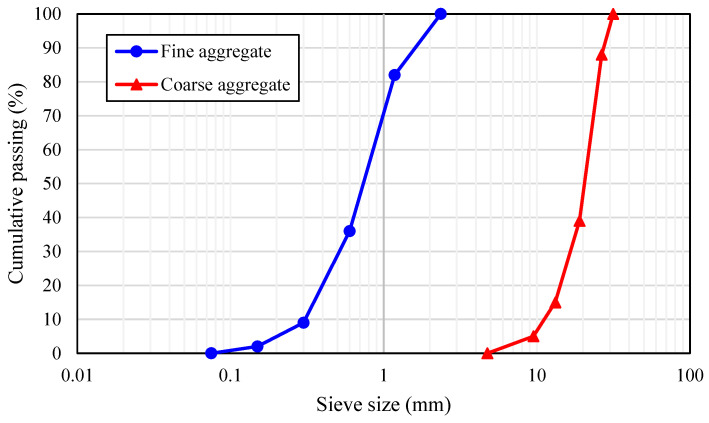
Particle size distribution of fine and coarse aggregate.

The concrete mixtures were prepared using Ordinary Portland Cement (OPC) with a strength grade of 42.5 MPa and a Blaine fineness of 375 m^2^/kg from Dujiangyan Lafarge Cement Co., Ltd. (Chengdu, China), conforming to GB 175-2023 [25]. Class F fly ash was sourced from Emei Hongyuan Resource Recycling Development Co., Ltd. (Emeishan, China), meeting GB/T 1596-2017 [26]. Fly ash was used as a supplementary cementitious material to partially replace cement and reduce hydration heat. The chemical compositions of the cement and fly ash are presented in Table 1. The fine aggregate was mechanically crushed sand (maximum particle size of 2.36 mm) in accordance with GB/T 14684-2022 [27], while the coarse aggregate was crushed granite stone (maximum particle size of 31.5 mm) conforming to GB/T 14685-2022 [28]. The particle size distributions of the coarse and fine aggregates are presented in Figure 1.

Potable water was used for mixing and curing. A constant water-to-binder ratio (w/b) of 0.42 was maintained across all mixtures to isolate the effect of the TRI. The chemical admixtures included a high-performance water-reducing admixture (WRA) to enhance workability and an air-entraining admixture (AEA) to improve durability.

The key variable in this study, TRI, was incorporated at dosages of 0%, 0.05%, 0.1%, and 0.15% by binder weight. It was sourced from Jinhua Xinsheng Zeolite Development Co., Ltd. (Jinhua, China). The TRIs used in this study were microencapsulated TRI, wherein the microcapsules consist of a polyethylene wax shell and a polysaccharide core. The polyethylene wax serves as a thermal-sensitive shell that melts at elevated temperatures to release the polysaccharide core, slowing down the hydration process and reducing heat generation. The TRI particle size ranges from 0.16 mm to 0.3 mm, and while larger TRI particles (closer to 0.3 mm) have a thicker encapsulation layer, leading to a slower release of hydration inhibitors, smaller TRI particles (closer to 0.16 mm) dissolve more rapidly, releasing hydration inhibitors at an earlier stage, ensuring uniform dispersion in the cementitious matrix. The hazardous substance detection results meet national standards (GB/T 17219-1998), indicating its safety for practical applications. Unlike conventional TRIs that dissolve and act immediately, microencapsulated TRIs release their active compounds gradually, leading to a prolonged suppression of hydration heat while minimizing negative effects on setting time and early age strength development [29]. Moreover, a control mix (C1) without TRIs was prepared, along with three additional mixes incorporating TRI dosages of 0.05%, 0.1%, and 0.15% (C2–C4). The total binder content was maintained constant to ensure comparability across all test groups. The detailed mix proportions for all test groups are presented in Table 2.

### 2.2. Mechanical Properties Test

In the experiment, the compressive strength, splitting tensile strength, and elastic modulus were measured according to Chinese Standard GB50010-2010 [30]. The size of the specimens was concrete cylinder with 100 mm diameter and 200 mm height for the compressive and splitting tensile strength test. A load was gradually applied until the specimen failed, and the compressive strength was recorded. A compressive and splitting tensile strength test was conducted on the 3rd, 7th, and 28th days to observe the change in concrete strength over time, which was crucial for evaluating maturity and predicting long-term performance. The elastic modulus test measures the deformation of a material under stress within the elastic range. In this experiment, the elastic modulus was calculated by measuring the strain of the specimen under static stress. The size of the specimens was 150 × 150 × 300 mm. The elastic modulus provides information about the elastic properties of the material, which is essential for structural design and vibration analysis. After pouring the concrete, the specimens are transferred to a controlled environment maintained at a constant temperature (20 ± 1 °C) and relative humidity (95 ± 5%) for standard curing.

### 2.3. Temperature Rise Test

To simulate temperature changes during actual construction conditions, three different-sized foam boxes were used. These foam boxes are chosen because they are highly common in daily life and frequently used for shipping and courier services. The first foam box measures 34 cm × 22 cm × 18 cm in size. The second foam box has dimensions of 45 cm × 29.5 cm × 26 cm. The third foam box is 53 cm × 37 cm × 29 cm in size. The foam boxes are named G1, G2, and G3 in descending order of size, as shown in Table 3.

**Figure 2:**
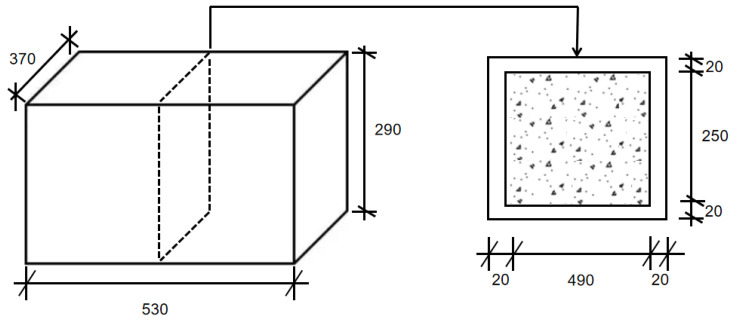
Schematic drawing of hydration temperature test setup (Unit: mm).

The temperature sensors used in this study were WRNT-010 high-precision thermocouples obtained from Jiangsu Weiyida Instrument Co., Ltd. (Taizhou, China). To ensure data reliability, all sensors were pre-calibrated using a standardized water bath calibration method, where temperature deviations remained within ±0.15 °C. Furthermore, to minimize measurement errors, each sensor was verified against a reference thermometer before and after testing. Temperature data were recorded at 30 s intervals, ensuring high-resolution monitoring of hydration heat evolution. However, it should be noted that this controlled laboratory setup does not fully replicate real construction conditions. In real construction site conditions, environmental temperature fluctuations, wind exposure, and humidity variations significantly influence heat dissipation, leading to differences in hydration heat evolution. Temperature sensors were affixed to the inner wall of the long side of the foam boxes using 52 mm wide, 200 mm long adhesive tape, ensuring that the sensor tip was positioned at the center of the inner wall for consistent temperature monitoring across all specimens. The entire setup was then completely sealed to prevent temperature interference from external environments, as shown in Figure 2. Concrete mixing was performed manually to maintain uniformity across all test groups. For the control mix, cement and aggregates were pre-mixed, followed by the addition of water, and the mixture was stirred for at least 180 s to ensure homogeneity. The water addition time was recorded as the reference point for temperature monitoring. For TRI-modified mixes, the inhibitor was incorporated at 0.05%, 0.1%, and 0.15% by binder weight, with precise dosages calculated accordingly. Dry components were first mixed evenly, followed by the addition of water, and the entire mixing process was completed within 10 min to ensure consistency.

The fresh concrete was immediately poured into the foam insulation boxes, which were then sealed with a lid and adhesive tape to prevent external heat exchange. Each temperature sensor was connected to a data acquisition system, and the corresponding sensor identification number was recorded at the time of water addition. The foam box identification number was matched with the assigned IoT temperature monitoring device to ensure accurate data tracking. To minimize thermal interference, a minimum spacing of 100 mm was maintained between the control and test specimens. The initial recorded temperature was maintained at 20 °C ± 2 °C, and the test continued until the recorded temperature dropped below 22 °C after reaching the peak hydration temperature. This method ensures reliable and repeatable measurement of hydration heat evolution, allowing for a direct comparison of the effects of different TRI dosages on temperature rise and dissipation patterns in medium-sized concrete specimens.

### 2.4. Microscopic Test

To further investigate the influence of microencapsulated TRIs on hydration suppression mechanisms and microstructural evolution, X-ray diffraction (XRD) (Dandong Haoyuan Instrument production Co., Ltd., Dandong, China) and scanning electron microscopy (SEM) (Carl Zeiss AG, Oberkochen, Germany) analyses were conducted. These microscopic techniques provide insight into the crystalline hydration products, hydration kinetics, and microstructural refinement induced by TRI incorporation.

SEM imaging was utilized to examine the microstructural morphology of cement hydration products in both the reference and TRI-modified concrete [31]. Samples were collected at designated curing ages, dried, vacuum-coated with gold, and analyzed under a high-resolution SEM at an accelerating voltage of 15 kV. XRD analysis was employed to identify crystalline hydration phases and evaluate the impact of TRIs on hydration product formation and dissolution rates. Diffraction patterns were obtained using a θ–2θ scanning mode in the range of 10–90°, with a step size of 0.02° and a scanning speed of 0.5°/min. The primary crystalline phases examined included calcium hydroxide (CH, portlandite), calcium silicate hydrate (C-S-H), unhydrated clinker phases (C_3_S, C_2_S), and sulfate-related phases (ettringite, monosulfate). Differences in peak intensities and shifts were analyzed to assess the effect of TRIs on hydration kinetics, particularly regarding the reduction in early stage CH crystallization, delayed C-S-H formation, and retention of unhydrated cement phases [32].

### 2.5. Quality Assurance

To ensure the accuracy and reliability of experimental data, rigorous quality assurance procedures were implemented throughout the study. Instrument calibration was conducted before each experimental run, with thermocouples for hydration temperature measurement verified against a reference thermometer with an accuracy of ±0.15 °C and the compression testing machine calibrated according to Chinese Standard GB50010-2010 [30]. Repeatability and reproducibility were ensured by performing each mechanical property test on three specimens, with reported values representing the mean and standard deviation, while hydration temperature data were collected using three independent thermocouples placed at different positions in the concrete mix to maintain consistency.

## 3. Test Results and Discussion

### 3.1. Compressive Strength

**Figure 3:**
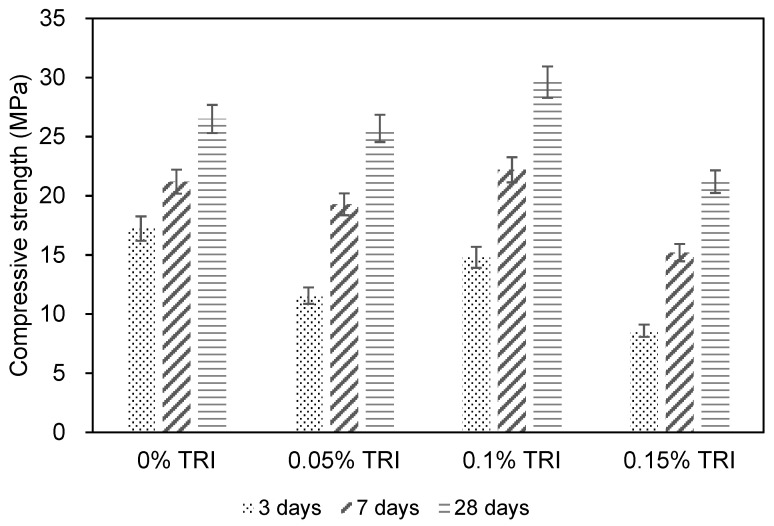
Compressive strength of concrete with different content of TRI.

Figure 3 shows the compressive strength development of concrete specimens with different TRI dosages (0%, 0.05%, 0.1%, and 0.15%) over curing ages of 3, 7, and 28 days. The reference concrete achieved a compressive strength of 26.5 MPa at 28 days, while the TRI-modified concretes exhibited variations in both early and later-stage strength development. At 3 days, the control mix C1 attained a compressive strength of 17.2 MPa, while C2, C3, and C4 exhibited strengths of 11.6 MPa, 14.8 MPa, and 8.6 MPa, respectively. This indicates that the inclusion of TRIs led to a reduction in early age strength, with the effect being more pronounced at higher dosages. This reduction is likely due to the inhibitory effect of the TRIs on the cement hydration process, wherein the microencapsulated TRIs release polysaccharides gradually as the polyethylene wax shell melts upon reaching a preset temperature threshold. This controlled-release mechanism forms a temporary adsorption layer on cement particle surfaces, delaying the hydration of tricalcium silicate (C_3_S) and dicalcium silicate (C_2_S) [33]. This controlled release mechanism slows down the initial hydration reaction, thereby reducing the early age strength. At 7 days, a similar trend was observed, with the control mix C1 achieving 21.2 MPa, while C2, C3, and C4 reached 19.3 MPa, 22.2 MPa, and 15.2 MPa, respectively. The strength difference between the control and TRI-modified specimens was less pronounced compared to the 3-day results, indicating that the hydration process in TRI-modified mixtures progressively accelerates over time as the hydration barriers are gradually dissolved. At 28 days, the compressive strength of the reference concrete C1 was 26.5 MPa, while C2, C3, and C4 achieved 25.7 MPa, 29.6 MPa, and 21.2 MPa, respectively. The C3 mix (0.1% TRI) surpassed the control mix, suggesting that moderate TRI dosages can improve long-term strength retention by promoting a more uniform and sustained hydration process. However, C4 (0.15% TRI) exhibited a noticeable reduction in 28-day strength (21.2 MPa), indicating that excessive TRI content may overly suppress hydration, leading to inadequate strength development. TRI content at 0.1% effectively reduce hydration heat while maintaining early age strength. Compared to chemical retarders such as urea, which has been reported to reduce compressive strength in self-consolidating concrete (SCC) [34], TRIs offer a more controlled hydration suppression mechanism. To validate the significance of differences between samples, an analysis of variance (ANOVA) was performed on the compressive strength results. The compressive strength increased with increasing TRI content up to 0.1%, after which a slight reduction was observed at 0.15%. ANOVA results indicate that TRI dosage had a statistically significant effect on compressive strength (*p* = 0.032 for 3 days, *p* = 0.045 for 7 days, and *p* = 0.018 for 28 days). This confirms that the observed differences are not due to random variations, but rather a direct effect of TRIs on hydration and strength development.

Based on the strength development trends, 0.1% TRI (C3) is identified as the optimal dosage, as it not only mitigates early age temperature rise but also results in the highest 28-day compressive strength (29.6 MPa). Lower dosages (0.05%) exhibit a milder effect but still slightly reduce early age strength, whereas higher dosages (0.15%) significantly delay hydration and compromise long-term strength. While the incorporation of microencapsulated TRIs effectively reduces hydration heat and mitigates thermal cracking, the early age strength reduction observed at higher TRI dosages (e.g., 0.15%) presents potential challenges in construction. In practical applications, early age strength is crucial for formwork removal, load-bearing capacity, and construction sequencing. A significant reduction in early compressive strength may delay formwork removal, extending the overall construction schedule, particularly in fast-paced projects such as high-rise buildings and precast concrete elements. For instance, conventional concrete formwork is often removed after 3–7 days, depending on the strength development. If TRI-modified concrete at 0.15% dosage exhibits significantly lower 3-day strength, it may require an extended curing period before formwork removal, which could increase labor and equipment costs. However, for applications where thermal cracking is a critical concern and immediate load-bearing capacity is not required, a controlled reduction in early age strength may be an acceptable trade-off for improved long-term durability. Additionally, in environments with higher ambient temperatures, TRIs can help regulate excessive heat accumulation, reducing thermal stress even if early age strength is slightly compromised. Thus, for practical applications where both temperature control and strength development are critical, a TRI dosage of 0.1% is recommended to achieve an optimal balance between hydration suppression and long-term performance in medium-sized concrete structures.

### 3.2. Splitting Tensile Strength

**Figure 4:**
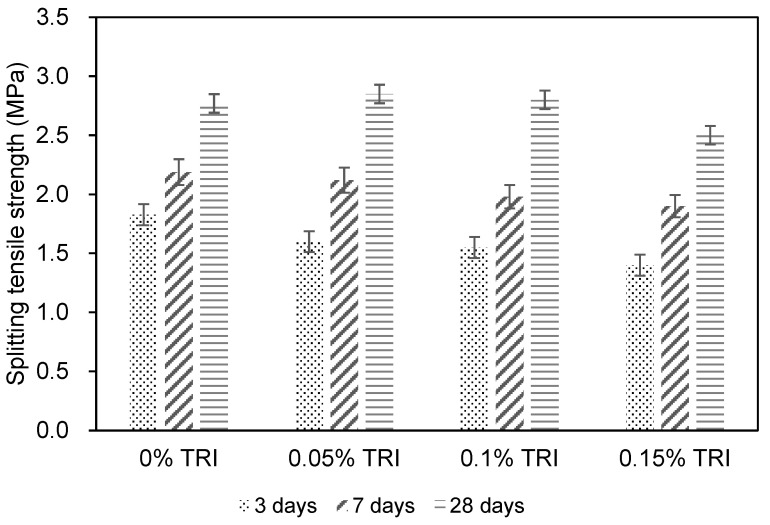
Splitting tensile strength of concrete with different content of TRI.

Figure 4 presents the development of splitting tensile strength for concrete specimens with different TRI dosages (0%, 0.05%, 0.1%, and 0.15%) at curing ages of 3, 7, and 28 days. The reference concrete C1 achieved a splitting tensile strength of 2.8 MPa at 28 days, while the TRI-modified concretes exhibited variations in both early and later-stage strength development. At 3 days, the control mix C1 attained a splitting tensile strength of 1.8 MPa, while C2, C3, and C4 exhibited strengths of 1.6 MPa, 1.6 MPa, and 1.4 MPa, respectively. This indicates that the incorporation of TRIs leads to a reduction in early age tensile strength, similar to the trend observed in compressive strength. The hydration–suppressing effect of TRI, which results in a delayed formation of hydration products, likely contributes to this initial strength reduction. At 7 days, the control mix reached 2.2 MPa, while C2, C3, and C4 measured 2.1 MPa, 2.0 MPa, and 1.9 MPa, respectively. The gap between the control and TRI-modified mixes narrowed, indicating that the hydration process gradually compensates for the initial strength delay as the encapsulated TRIs continue to dissolve and release active components. At 28 days, the reference concrete C1 and C3 both achieved 2.8 MPa, whereas C2 exhibited a slightly higher value of 2.9 MPa, and C4 had the lowest strength at 2.5 MPa. The similar or even improved long-term tensile strength in C2 and C3 suggests that a moderate TRI dosage (≤0.1%) does not negatively impact the final tensile strength, as hydration catches up over time. However, at 0.15% TRI dosage (C4), a clear reduction in tensile strength is observed, indicating that excessive hydration suppression may compromise the formation of tensile-resistant structures, leading to a weaker tensile response.

The splitting tensile strength of concrete plays a crucial role in determining its resistance to thermal cracking, as tensile stresses develop when temperature gradients cause differential expansion and contraction within the structure. Concrete typically has low tensile strength, making it susceptible to thermal stress-induced cracking, especially during early age hydration. The C2 (0.05%) and C3 (0.1%) mixtures exhibited similar or slightly improved 28-day tensile strength compared to the control, suggesting good crack resistance. However, C4 (0.15%) had a notably lower splitting tensile strength, implying that an excessive TRI dosage may reduce the concrete ability to resist tensile stress. While this dosage effectively lowers peak hydration temperature, it may also increase thermal cracking susceptibility due to the reduced tensile capacity of the material. If thermal stresses exceed the tensile strength, cracking is more likely to occur, especially in restrained structural elements such as slabs and beams. Considering both hydration temperature control and mechanical performance, 0.05–0.1% TRI (C2 and C3) is the most effective dosage range, as it successfully mitigates early age hydration heat while maintaining or slightly improving the long-term tensile strength and crack resistance. Conversely, a 0.15% TRI dosage (C4) is not recommended, as it significantly lowers tensile strength and could increase susceptibility to thermal cracking, which is a key concern in temperature-controlled concrete applications. This suggests that an optimal TRI dosage (≤0.1%) should be selected to achieve effective thermal cracking mitigation without compromising mechanical integrity.

### 3.3. Elastic Modulus

**Figure 5:**
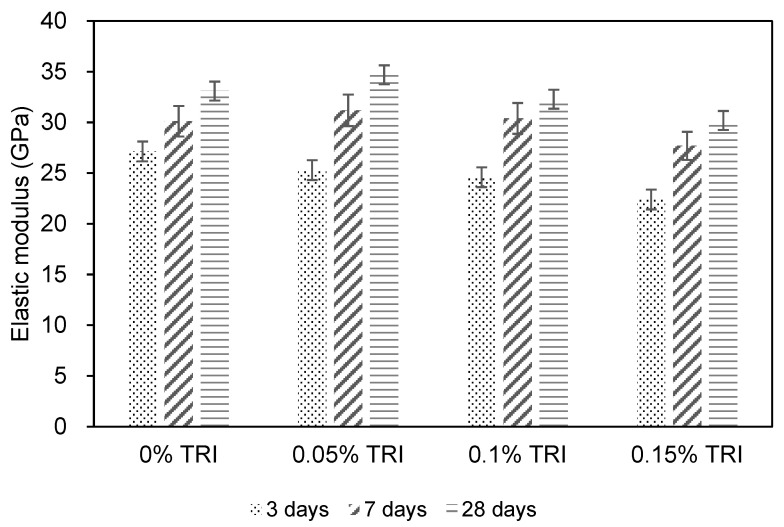
Elastic modulus of concrete with different contents of TRI.

Figure 5 illustrates the elastic modulus development of concrete specimens incorporating different TRI dosages (0%, 0.05%, 0.1%, and 0.15%) over curing periods of 3, 7, and 28 days. The reference concrete C1 achieved an elastic modulus of 33.1 GPa at 28 days, while the TRI-modified concretes exhibited varied trends in stiffness development, influenced by the hydration suppression effect. At 3 days, the elastic modulus of the control mix C1 was 27.1 GPa, while C2, C3, and C4 exhibited values of 25.3 GPa, 24.6 GPa, and 22.4 GPa, respectively. Similar to the trends observed in compressive and tensile strength, the addition of TRIs resulted in a notable reduction in early age stiffness, which is attributed to the delayed hydration reaction caused by the microencapsulated TRI. The controlled release of hydration inhibitors slows down the early formation of hydration products, leading to lower initial stiffness in the TRI-modified mixes [35]. At 7 days, the control mix reached 30.1 GPa, while C2, C3, and C4 measured 31.2 GPa, 30.4 GPa, and 27.7 GPa, respectively. The difference between the control and TRI-modified specimens was reduced, suggesting that the hydration process had advanced sufficiently in the TRI mixes, partially recovering stiffness. At 28 days, the elastic modulus of the reference mix C1 was 33.1 GPa, while C2 exhibited the highest value of 34.7 GPa, followed by C3 at 32.3 GPa, and C4 at 30.2 GPa. The results indicate that moderate TRI dosages (0.05–0.1%) slightly improve the long-term stiffness, while excessive TRI dosage (0.15%) leads to a lower modulus, likely due to the prolonged inhibition of hydration, affecting the formation of a dense microstructure.

Elastic modulus is a critical parameter in concrete structures, as it governs deformation resistance and stress distribution under loading. A higher elastic modulus generally correlates with better crack resistance and reduced long-term shrinkage, which is crucial in mitigating thermal stress-induced cracking. The C2 (0.05%) and C3 (0.1%) specimens exhibited comparable or slightly improved elastic modulus values at 28 days, indicating that these TRI dosages effectively balance hydration suppression with microstructure densification. However, C4 (0.15%) exhibited a lower modulus, suggesting that excessive TRI content may negatively impact stiffness, increasing the likelihood of deformation and cracking under restrained conditions. Based on the observed trends, 0.05–0.1% TRI (C2 and C3) is recommended, as it effectively reduces early age temperature rise while maintaining or slightly improving the long-term elastic modulus, thereby enhancing overall structural stability and crack resistance. Conversely, 0.15% TRI (C4) is not recommended, as it significantly lowers stiffness, which could increase the risk of cracking and deformation in temperature-sensitive concrete applications.

### 3.4. Effect of TRIs on Temperature Rise

**Figure 6:**
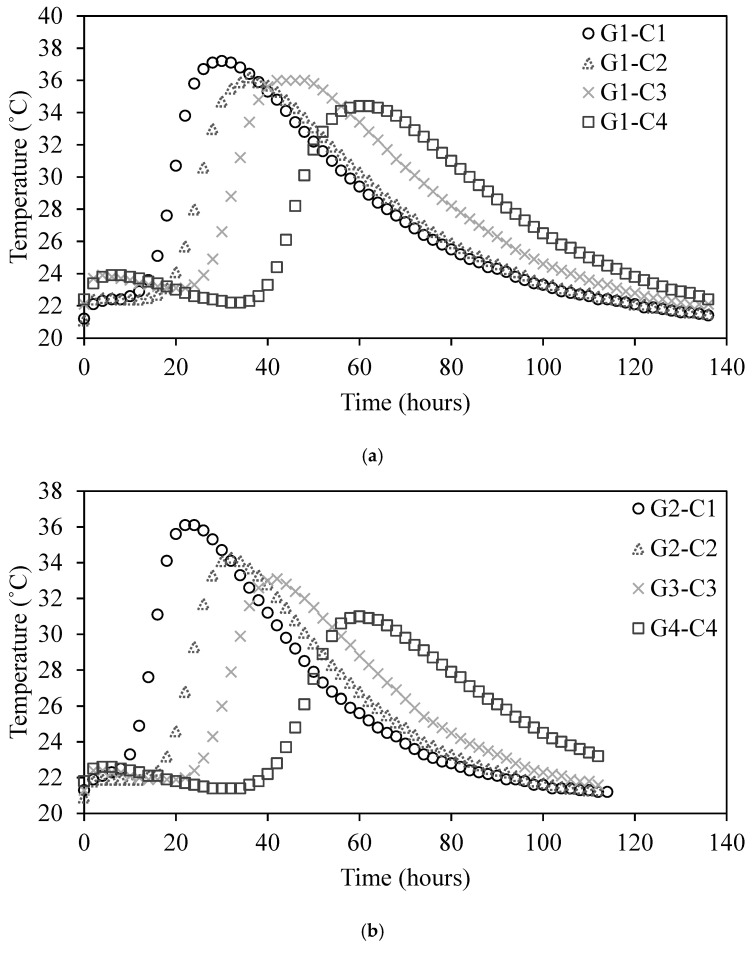

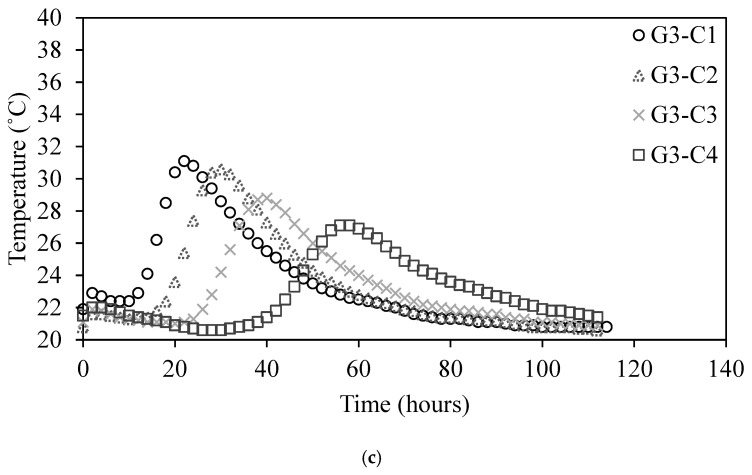
Hydration temperature of the (**a**) G1 group, (**b**) G2 group, and (**c**) G3 group.

To investigate the influence of TRIs on the hydration temperature of the concrete, Figure 6 shows the temperature curves for the G1, G2, and G3 groups. The changes in the maximum hydration temperature of each group of concrete as the admixture dosage increased can be observed. In the G1 set of experiments, through careful observation and data recording, it was found that, with increasing TRI content, the maximum hydration temperature of the concrete gradually decreased [36]. Specifically, when the admixture content was 0%, 0.05%, 0.1%, and 0.15%, the maximum hydration temperatures of the concrete were 37.2 °C, 36.2 °C, 36 °C, and 34.5 °C, respectively. Compared with those of the baseline concrete, the maximum hydration temperatures decreased by approximately 2.7%, 3.2%, and 7.3%, respectively. Through calculations and data analysis, as the TRI content increased, the hydration rate of the concrete gradually decreased.

Figure 6 also shows that the maximum hydration temperature was reached. For specimens with admixture contents of 0%, 0.05%, 0.1%, and 0.15%, the peaks appeared at 28.5 h, 34.4 h, 43.3 h, and 59.4 h, respectively. It is evident that the time was delayed. This suggests that the controlled release mechanism of the microcapsules extends the hydration process, shifting the temperature peak to a later stage. The hydration temperature in Figure 6c shows that 0.1% TRI effectively reduces peak hydration temperature by 2.3 °C. In contrast, 0.05% TRI shows only a minor reduction of 0.4 °C, which may be insufficient for controlling thermal cracking in medium-sized elements. At 0.15% TRI, hydration suppression is too strong, leading to prolonged setting times that could delay construction schedules.

### 3.5. Effect of Size on Temperature Rise

**Figure 7:**
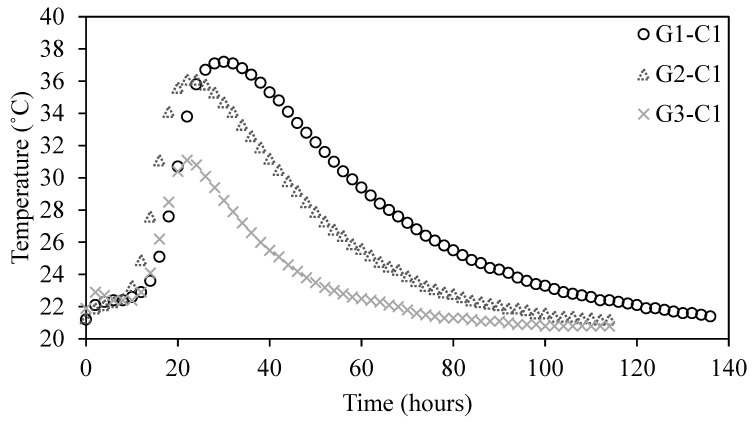
Temperature curves of reference concrete with different dimensions.

A comparison of the hydration temperature evolution for G1, G2, and G3 concrete specimens in sealed foam insulation boxes is presented in Figure 7. The results indicate that larger concrete volumes exhibit higher peak hydration temperatures and delayed temperature peaks due to internal heat retention effects. Specifically, the maximum hydration temperatures for G1, G2, and G3 are 37.2 °C, 36.1 °C, and 31.1 °C, respectively. Additionally, the time to reach the peak temperature follows a similar trend, occurring at 28.5 h for G1, 21.4 h for G2, and 21.3 h for G3.

Since the experiments were conducted in sealed foam boxes, external heat exchange was minimized, meaning that the primary factor influencing temperature differences was internal heat generation and retention. In larger specimens (G1), the lower surface-area-to-volume ratio led to reduced heat dissipation, causing a more pronounced accumulation of hydration heat and a delayed peak temperature. In contrast, smaller specimens (G3) had a higher relative surface area, which allowed for faster internal heat dispersion, leading to a lower peak temperature and an earlier occurrence of the hydration peak.

Furthermore, in the insulated environment, heat generated during hydration remained trapped within the specimens, leading to a more uniform internal temperature gradient but also prolonging the heat dissipation process. This effect was more evident in larger specimens, where the higher cumulative heat release intensified the thermal buildup, increasing the risk of thermal stress and cracking if not properly managed [37].

These findings emphasize the importance of temperature control strategies in medium-sized concrete elements, particularly when external cooling is not feasible. The results further support the application of temperature rise inhibitors to regulate hydration heat, preventing excessive temperature differentials and ensuring long-term structural integrity in temperature-sensitive concrete applications.

### 3.6. Microscopic Analysis

**Figure 8:**
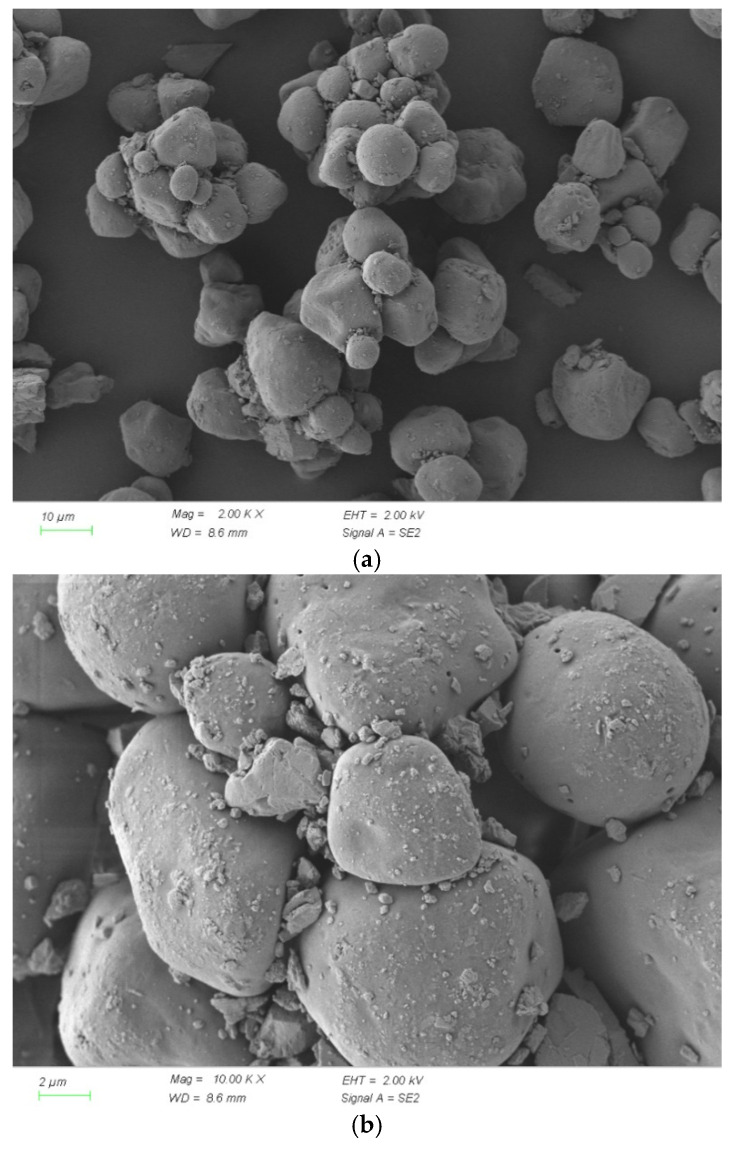
SEM of TRIs at different scales (**a**) 10 μm; (**b**) 2 μm.

The SEM micrographs provide further insights into the physical characteristics of microencapsulated TRI, as shown in Figure 8. These images confirm that the TRI microcapsules exhibit a spherical morphology with a relatively smooth surface, which facilitates controlled release during hydration. The well-defined microcapsule structure ensures that the hydration inhibitors are gradually released rather than dissolved immediately, enabling sustained temperature regulation.

While these SEM images focus on the morphology of TRI particles, further microstructural analysis of TRI-modified concrete is needed to evaluate their interaction with the cement matrix and the resulting hydration suppression mechanisms. Previous research suggests that microencapsulated TRIs can form a temporary hydration barrier by adsorbing onto cement particles, thereby delaying the reaction of tricalcium silicate (C_3_S) and tricalcium aluminate (C_3_A). This controlled release mechanism moderates hydration kinetics, preventing rapid heat accumulation and reducing the risk of early age thermal cracking.

From an engineering perspective, a TRI dosage of 0.1% appears to be the most effective in balancing hydration suppression and microstructural development. This dosage achieves sufficient heat reduction while ensuring that hydration progresses at a controlled rate, leading to favorable mechanical properties. In contrast, higher TRI dosages, such as 0.15%, may induce excessive hydration delays, potentially compromising early age performance. Thus, the results demonstrate that microencapsulated TRIs is a viable strategy for mitigating hydration heat in medium-sized concrete structures, offering a practical solution for reducing thermal cracking risk while maintaining structural integrity.

**Figure 9:**
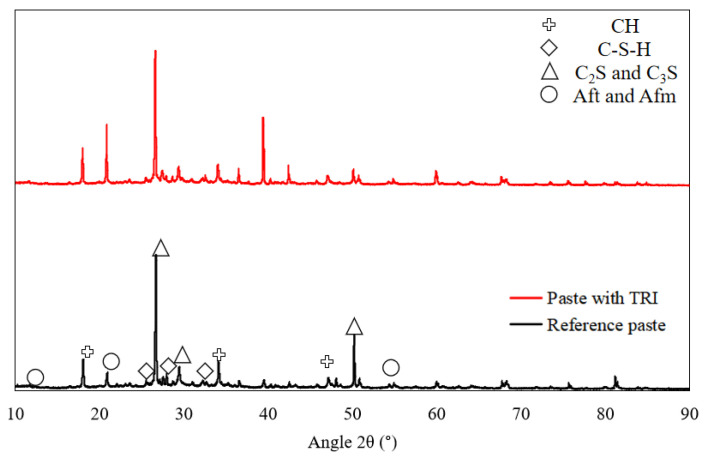
XRD analysis of paste with and without TRI.

Moreover, the crystalline phase composition of the reference cement paste (without TRI) and TRI-modified cement paste was analyzed using X-ray diffraction (XRD), as shown in Figure 9. The diffraction patterns provide insights into the influence of the microencapsulated TRIs on cement hydration, particularly in the evolution of key hydration products such as calcium hydroxide (CH), calcium silicate hydrate (C-S-H), and unhydrated clinker phases (C_3_S, C_2_S). Since C-S-H is predominantly amorphous, its presence in XRD is inferred rather than directly detected as distinct peaks.

Distinct diffraction peaks corresponding to calcium hydroxide (CH, portlandite) at 18.1°, 34.1°, and 47.1° were observed in the reference cement paste. These peaks represent the direct byproducts of C_3_S and C_2_S hydration, which contribute to the alkalinity of the pore solution and influence microstructural development. In the TRI-modified cement paste, the intensity of CH peaks was significantly reduced, indicating a delayed hydration process due to the controlled release of TRI components. This finding is consistent with the hydration temperature evolution trends, where the incorporation of TRIs resulted in a lower peak temperature and a prolonged heat release period, further supporting the hypothesis that TRIs slow down early stage cement hydration [38].

The formation of calcium silicate hydrate (C-S-H), the primary hydration product responsible for strength development, is typically detected as a broad diffraction hump between 20 and 35° due to its low crystallinity. However, in XRD analysis, C-S-H does not exhibit sharp diffraction peaks, and its identification relies on indirect interpretation rather than direct detection. In the TRI-modified cement paste, this broad peak appeared wider and slightly shifted, suggesting that the TRIs influenced the nucleation and growth of C-S-H, leading to a more homogeneous and refined gel structure. This observation is consistent with the SEM analysis, which revealed a denser microstructure with improved pore refinement in TRI-modified specimens. The suppression of early CH formation, combined with a gradual C-S-H evolution, contributes to a more uniform hydration process, reducing the risk of thermal stress-induced cracking. Delayed C-S-H formation at early ages corresponds to lower early age strength but potentially enhances later-age strength. The hydration inhibition effect observed in XRD aligns with the compressive strength trend. The XRD and hydration temperature results further indicate that the incorporation of TRIs enhances long-term durability by modifying hydration kinetics. The delayed hydration leads to a gradual development of C-S-H, reducing internal stresses and improving resistance to sulfate attack, carbonation, and chloride penetration. Additionally, the observed reduction in CH crystallization suggests that the hydration process results in a more stable cement matrix, which is crucial for long-term mechanical performance.

Peaks associated with unhydrated tricalcium silicate (C_3_S) and dicalcium silicate (C_2_S) at 29.3°, 32.2°, and 50.1° were observed in both samples. However, the intensity of these peaks was relatively stronger in the TRI-modified cement paste, indicating that the hydration process was inhibited, leaving a higher proportion of unreacted cement particles at the same curing age. This finding further corroborates the delayed hydration kinetics observed in the temperature monitoring tests. The presence of higher residual clinker phases suggests that TRIs effectively reduce the rate of cement dissolution, leading to a more extended hydration reaction that continues beyond early age curing.

Diffraction peaks corresponding to ettringite (Aft) at ~11.3° and 22.9° were identified in both concrete samples, indicating the reaction of tricalcium aluminate (C_3_A) with sulfate sources. However, in the TRI-modified cement paste, these peaks were less pronounced, suggesting that TRIs affected sulfate reaction kinetics, possibly delaying the transformation of ettringite into monosulfate (Afm at 56.5°). The reduced intensity of ettringite peaks may be associated with the adsorption of TRI components on cement particles, which limits ion mobility and initial hydration reactions.

The combined XRD, hydration temperature, and SEM analyses reveal that the hydration suppression effect of the microencapsulated TRIs follows a controlled-release mechanism that gradually influences the hydration process. Initially, the TRIs remain physically intact within the cement paste, encapsulated by its polymeric shell. As the cement hydrates, the encapsulated TRIs slowly dissolve, releasing hydration inhibitors that adsorb onto the surfaces of cement particles. This adsorption forms a temporary hydration barrier, restricting the dissolution of tricalcium silicate (C_3_S) and dicalcium silicate (C_2_S), which in turn reduces the rate of calcium hydroxide (CH) formation. The XRD results confirm this by showing a lower CH peak intensity in TRI-modified cement paste compared to the reference mix.

This hydration delay affects the formation and growth of calcium silicate hydrate (C-S-H), as indicated by the subtle and slightly shifted XRD diffraction hump in the 20–35° range. Since C-S-H is predominantly amorphous, its presence in XRD data is inferred rather than directly observed as distinct peaks. The gradual development of C-S-H contributes to a more refined and homogeneous microstructure, as observed in SEM images. Additionally, the retention of unhydrated clinker phases (C_3_S and C_2_S) in XRD analysis further corroborates the slower reaction kinetics induced by TRI. Moreover, the presence of sulfate-based hydration products such as ettringite (Aft) and monosulfate (Afm) appears to be slightly altered, with weaker diffraction peaks in the TRI-modified mix, suggesting a delayed aluminate reaction due to limited ion diffusion.

The hydration temperature evolution data reinforce this suppression mechanism. TRI incorporation lowers the peak hydration temperature and delays the time to reach peak heat release, effectively spreading out heat generation over an extended period. This controlled heat evolution minimizes sudden temperature gradients, reducing the likelihood of thermal stress-induced cracking in medium-sized concrete elements. Additionally, the foam box insulation environment used in the experiments ensured minimal external heat exchange, highlighting that the observed effects are predominantly attributed to the internal hydration process rather than environmental influences.

The findings demonstrate that microencapsulated TRIs provide an effective means of regulating hydration temperature in TRI-modified cementitious systems, particularly in applications where external cooling measures are impractical. The ability of TRIs to moderate early age heat generation while allowing for continued hydration over time makes it a viable solution for preventing temperature-induced cracking in structures with constrained thermal dissipation.

Based on the observed hydration behavior, a TRI dosage of 0.1% is recommended as the optimal balance between hydration suppression and mechanical performance. At this dosage, the hydration temperature peak is effectively reduced without significantly compromising early age strength development. The TRI-modified cement paste at 0.1% dosage exhibits a well-refined microstructure, contributing to improved long-term durability and reduced thermal stresses. In contrast, a higher dosage of 0.15% TRI results in excessive hydration suppression, leading to prolonged reaction times and potential delays in strength gain, which may not be ideal for structural applications requiring early age load-bearing capacity.

## 4. Conclusions

This study systematically investigated the effect of microencapsulated temperature rise inhibitors on hydration heat evolution, microstructural development, and mechanical properties of medium-sized concrete structures. The findings of this study are as follows:

(1) Microstructural analysis via XRD and SEM revealed that the hydration suppression effect of TRIs follows a controlled-release mechanism. The encapsulated TRIs gradually dissolve over time, forming a temporary hydration barrier on cement particles, thereby slowing CH crystallization and altering C-S-H gel development. This mechanism results in reduced early age heat accumulation, effectively mitigating thermal stress-induced cracking.
(2) Mechanical performance tests indicated that TRI incorporation slightly reduces early age compressive and tensile strength due to delayed hydration. However, strength recovery was observed over time, with TRI-modified concrete achieving comparable long-term mechanical properties to the reference mix. Notably, a TRI dosage of 0.1% provided the optimal balance, significantly reducing hydration temperature while maintaining acceptable strength development. In contrast, higher dosages (0.15%) excessively suppressed hydration, leading to prolonged setting time and potential strength limitations.
(3) From an engineering perspective, these findings highlight the potential of TRIs as a practical strategy for controlling hydration temperature in medium-sized concrete structures, particularly in applications where external cooling measures are limited. A 0.1% TRI dosage was found to be the optimal balance between hydration heat suppression and mechanical integrity, reducing peak temperature rise while maintaining adequate early age strength. Using more than 0.1% TRI can lead to prolonged setting times and reduced strength, whereas less than 0.1% TRI may be insufficient in controlling hydration heat effectively. The controlled-release mechanism of TRIs ensures gradual hydration suppression, which minimizes thermal stress differentials and enhances long-term durability.
(4) While its current production cost is higher than traditional retarders, its ability to mitigate thermal stress and improve long-term durability may offset the initial cost. Further research should focus on cost-effective encapsulation methods to enhance the competitiveness of TRIs in commercial concrete applications. Future studies should also incorporate field trials on real construction site conditions to better understand the performance of TRIs under practical conditions.

## Figures and Tables

**Table 1:** Chemical compositions of cement and fly ash.

Chemical Oxides	SiO_2_	CaO	Al_2_O_3_	SO_3_	Fe_2_O_3_	K_2_O	MgO	Na_2_O	TiO_2_
OPC	20.31	65.5	4.8	2.1	4.99	0.4	1.3	0.15	0.39
FA	46.59	4.98	38.52	0.66	3.93	0.66	0.96	0.2	1.69

**Table 2:** Mixture proportions.

Sample ID	Cement	Fly Ash	Fine Aggregate	Coarse Aggregate	WRA	AEA	Water	TRI
C1	280	150	675	1148	5.16	2.15	140	0
C2	280	150	675	1148	5.16	2.15	140	0.05%
C3	280	150	675	1148	5.16	2.15	140	0.1%
C4	280	150	675	1148	5.16	2.15	140	0.15%

**Table 3:** Dimension of foam boxes (mm).

Group ID	Length	Width	Height
G1	530	370	290
G2	450	295	260
G3	340	220	180

## Data Availability

The original contributions presented in this study are included in the article. Further inquiries can be directed to the corresponding authors.
